# Stabilization of hypoxia inducible factor by cobalt chloride can alter renal epithelial transport

**DOI:** 10.14814/phy2.13531

**Published:** 2017-12-21

**Authors:** Subhra Nag, Andrew Resnick

**Affiliations:** ^1^ Department of Biology, Geology and Environmental Sciences Cleveland State University Cleveland Ohio; ^2^ Department of Physics Cleveland State University Cleveland Ohio; ^3^ Center for Gene Regulation in Health and Disease Cleveland State University Cleveland Ohio

**Keywords:** Cyst, electrophysiology, hypoxia, ischemia, kidney epithelia

## Abstract

Given the importance of the transcriptional regulator hypoxia‐inducible factor‐1 (HIF‐1) for adaptive hypoxia responses, we examined the effect of stabilized HIF‐1*α* on renal epithelial permeability and directed sodium transport. This study was motivated by histological analysis of cystic kidneys showing increased expression levels of HIF‐1*α* and HIF‐2*α*. We hypothesize that compression induced localized ischemia‐hypoxia of normal epithelia near a cyst leads to local stabilization of HIF‐1*α*, leading to altered transepithelial transport that encourages cyst expansion. We found that stabilized HIF‐1*α* alters both transcellular and paracellular transport through renal epithelial monolayers in a manner consistent with secretory behavior, indicating localized ischemia‐hypoxia may lead to altered salt and water transport through kidney epithelial monolayers. A quantity of 100 *μ*mol/L Cobalt chloride (CoCl_2_) was used acutely to stabilize HIF‐1*α* in confluent cultures of mouse renal epithelia. We measured increased transepithelial permeability and decreased transepithelial resistance (TER) when HIF‐1*α* was stabilized. Most interestingly, we measured a change in the direction of sodium current, most likely corresponding to abnormal secretory function, supporting our positive‐feedback hypothesis.

## Introduction

Kidney cysts are characterized by epithelial‐lined fluid filled sacs where the fluid is stagnant and loaded with cytokines (Calvet and Grantham 2001; Cowley et al. 2001; Grantham 2001; Zheng et al. 2003). By compressing neighboring tissue, cyst expansion may create localized ischemia‐hypoxia (Bernhardt et al. 2007; Belibi et al. 2011). Cyst expansion is associated with transepithelial fluid secretion, in contrast to the normal absorptive function (Sullivan et al. 1998). HIF‐1*α* has been shown to promote cyst expansion through calcium activated chloride secretion (Buchholz et al. 2013). In order for kidney cysts to expand, both active (transcellular) ion transport as well as passive (paracellular) transport may be altered, possibly converting a normally absorptive epithelium to a pathological secretory epithelium. It has been shown that cultured normal kidney cells (MDCK and primary cultures of human cortical cells) can be induced to form microcysts via the cAMP pathway ((Mangoo‐Karim et al. 1989) and reviewed in (Sullivan et al. 1998)). Importantly, those studies demonstrated that abnormalities in either PKD1 or PKD2 genes are not required to either induce cyst formation or fluid secretion by renal epithelial cells: the genetics of kidney cyst disease are independent of altered epithelial transport.

We hypothesize that, within the context of cystic kidney disease (PKD), stabilized Hypoxia Inducible Factor (HIF) alters renal epithelial function and encourages cyst expansion through altered salt and water transport. Because the genetic defects underlying PKD do not directly alter epithelial transport, we base our hypothesis on histological analysis of cystic kidneys showing increased levels of HIF‐1*α*, HIF‐2*α*, and increased serum level of Erythropoietin (EPO) (Chandra et al. 1985; Zeier et al. 1996; Bernhardt et al. 2007; Belibi et al. 2011). In response to hypoxic conditions, HIF‐1*α* is stabilized and translocates to the nucleus, resulting in the elevation of several effector molecules, such as EPO and vascular endothelial growth factor (VEGF) (Wang and Semenza 1993; Semenza 1999). In the presence of normoxia, HIF‐1*α* undergoes hydroxylation by prolyl hydroxylase and hydroxylated HIF‐1*α* undergoes proteasomal degradation (Ivan et al. 2001). CoCl_2_ is well known for its ability to stabilize hypoxia inducible factor 1*α* (HIF‐1*α*) (Kaelin 2002; Yuan et al. 2003). CoCl_2_ was shown to stabilize HIF‐1*α* by preventing hydroxylation of HIF‐1*α* (Wang and Semenza 1993; Yuan et al. 2003).

Prior reports have demonstrated changes to transepithelial sodium transport due to fluid flow (Sullivan et al. 1998; Resnick and Hopfer 2007), so we also wished to determine if flow‐driven transport alterations somehow interact (positively or negatively) with transport changes caused by stabilized HIF. The rationale for this measurement is provided by the fluid flow alteration within and in the vicinity of a cyst: flow is slowed or even stopped. Increased imbibition of water is becoming recognized as a viable treatment for PKD (Wang et al. 2013), and while attention has focused on the dilution of the antidiuretic hormone arginine vasopressin (AVP), increased water intake also results in higher tubular flow rates, supporting our hypothesis that fluid flow can impact PKD disease progression.

Our experimental system consisted of a cultured kidney epithelial cell line originally microdissected from the cortical collecting duct (mCCD) of an Immortomouse^®^, chosen because of the particularly simple salt and water transport system. The kidney collecting duct is known to “fine tune” salt and water balance in the body, contributing 0.5% the final salt resorption (Fauci 2008; Resnick 2011). In the collecting duct epithelium, sodium ions enter cells through epithelial sodium channels (ENaC) localized to the apical membrane and exit the basolateral side via the transporter protein sodium‐potassium‐ATPase (Na^+^K^+^ATPase) (Garty and Palmer 1997; Fauci 2008). Apical and basolateral K^+^ channels act to clear K^+^ build up within the cells (Garty and Palmer 1997; Fauci 2008). Net movement of positive Na^+^ ions from urine to blood creates an ionic gradient, driving paracellular transport of negatively charged ions, principally Cl^−^, in the same direction(Garty and Palmer 1997; Sullivan et al. 1998; Fauci 2008).

Using confluent monolayers of differentiated mouse kidney epithelia, we experimentally stabilized HIF by addition of CoCl_2_ and measured causal changes in transepithelial transport of sodium and water. We then compared HIF‐stabilized altered transepithelial sodium transport in the presence or absence of flow. Here, we report that applying CoCl_2_ at a concentration commonly used for HIF stabilization results in altered transcellular and paracellular transport of salt and water in otherwise normal renal epithelial cells. We also report that application of fluid flow blunts the alteration of salt and water transport caused by stabilized HIF.

Because directed transport of salt and water occurs simultaneously through both paracellular and transcellular paths, single electrophysiological measurements of transepithelial currents are unable to distinguish between the two paths. Thus, we performed two measurements, one of the transepithelial permeability using FITC‐labeled Dextran molecules (3 kDa and 70 kDa molecular weights) as paracellular tracers, and one of the transepithelial amiloride‐sensitive sodium current. Two molecular weights of FITC‐Dextran were used because 3 kDa FITC‐dextran may also be transported via transcellular pathways due to increased fluid‐phase transcytosis (Matter and Balda 2003; Balda and Matter 2007). Because the paracellular pathway shows a much stronger size selectivity than transcytosis (Matter and Balda 2003), comparing changes of the two molecular weight FITC‐Dextran permeabilities allows us to distinguish between changes to paracellular transport and changes to transcellular transport.

In summary, to better understand the transport mechanisms for kidney cyst expansion, we performed a series of measurements on cultured renal epithelia to examine what changes in transepithelial salt and water transport may occur when HIF is stabilized and if these changes are fluid flow‐dependent.

## Methods

### Cell culture

Experiments were carried out on a mouse cell line obtained by microdissection from the cortical collecting duct (mCCD 1296 (d)) of a heterozygous offspring Immortomouse^®^ carrying a transgene, temperature‐sensitive SV40 large T antigen under the control of an interferon‐*γ* response element (Jat et al. 1991). Cells were cultured on suspended membrane cell culture inserts. The mCCD cell line retains the major phenotypic characteristics observed in primary cortical collecting duct cultures including contact inhibition, tight junction formation, cell polarity, and directed transport of salt and water.

mCCD cells were grown to confluence under normoxia incubation conditions of 33°C, 5% CO_2_, during differentiation cells were incubated at 39°C, 5% CO_2_. The growth medium was prepared by combining the following media constituents (final concentrations): Dulbecco's Modified Eagle Medium (DMEM) w/o glucose and Ham's F12 (at a ratio of 1:1), 5 mg/mL transferrin, 5 mg/mL insulin, 10 mg/mL epithelial growth factor (EGF), 4 mg/mL dexamethasone, 15 mmol/L 4‐(2‐hydroxyethyl)‐ 1‐piperazineethanesulfonic acid (HEPES), 0.06 w/v% NaHCO_3_, 2 mmol/L L‐glutamine, 10 ng/mL mouse interferon‐*γ*, 50 *μ*mol/L ascorbic acid 2‐ phosphate, 20 nmol/L selenium, 1 nmol/L 3,39,5‐triiodo‐L‐thyronine (T3), and 5% fetal bovine serum (FBS). Cells were first expanded on collagen coated 30 mm diameter suspended membrane cell culture inserts (Millipore PICM03050, area= 4.71 cm^2^), inside a 6‐well plate (Falcon# 35116). After a sufficient quantity of cells were obtained, cells were passaged (1:3 dilution) onto 12 mm diameter suspended membrane cell culture inserts (Millipore PICM 01250, area = 1.13 cm^2^), within a 24‐well plate (Falcon# 353047), grown to confluence and allowed to differentiate resulting in a ciliated monolayer of epithelial cells exhibiting directed salt and water transport. For differentiation, growth factors (insulin, EGF, and interferon‐*γ*) were omitted from the culture media and additionally, FBS was omitted from the apical media.

**Figure 1:**
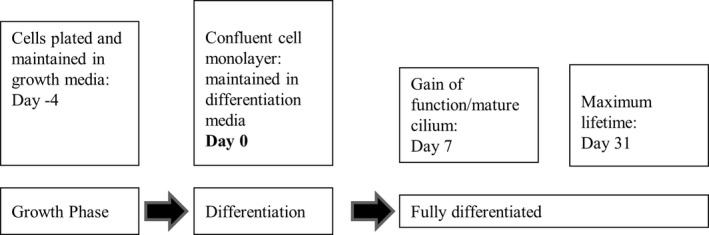
Cell culture timeline.

Figure 1 presents a schematic timeline of our experiments. Day 0 is defined as the starting point, when confluent monolayers were switched to differentiation conditions. Upon entering differentiation culture conditions, mCCD monolayers develop functional outputs over time. Directed sodium transport commences and a functional cilium emerges after approximately 5 days. mCCD monolayers are considered fully differentiated after 7–10 days, and remain viable for an additional 24 days. Throughout the experiment, the apical amount of medium was restricted (100 *μ*L), so that the thin fluid layer could allow sufficient oxygen diffusion (Resnick 2011).

### Application of fluid flow

Fluid flow has been shown to alter electrophysiological properties of renal epithelia (Resnick 2011). Cultures exposed to fluid flow were placed on an orbital shaker (Resnick 2011). Fluid flow was generated by operating the orbital plate shaker **(**IKA MS3 digital) at 4.2 Hz.

### Fluorescein isothiocyanate (FITC)‐Dextran Permeability Assay

Two molecular weight FITC‐Dextran molecules (3 kDa and 70 kDa), anionic, D 3305 and D 1823 (Molecular Probes, Life Technologies) in Hanks’ Balanced Salt Solution (HBSS), 1X (21‐023‐CM, Cellgro, Mediatech) were used to measure the transepithelial permeability. Molecular Probes specifies that the specific lots consist of 1 mole FITC per mole of Dextran (3 kDa, Lot# 1709577 and 1485224) and 7 moles FITC per mole Dextran (70 kDa, Lot # 1583589). For both molecular weights, the stock concentration (120 *μ*mol/L) was serially diluted to eight different concentrations, ranging from 120 *μ*mol/L to 120 × 10^−8^ *μ*mol/L. To generate a fluorescence intensity/FITC concentration curve, four replicates of 100 *μ*L solution at various concentrations were dispensed into a 96‐well plate and fluorescence values were measured using a fluorescence plate reader (Perker Elmer Life Sciences/Wallac Victor Multilabel Counter and Wallac 1420 Manager software) using 490 nm excitation and 530 nm emission. Calibration curves were later used to determine the FITC concentrations from the fluorescence intensity values during permeability experiments.

To perform FITC‐Dextran permeability measurements on our cultures, cell culture inserts were gently washed thrice with HBSS buffer to remove any media. Then, 100 *μ*L of FITC‐dextran in HBSS buffer was added to the apical side and 1 mL of HBSS buffer was added to the basolateral side of the cell culture inserts. A quantity of 100 *μ*L media samples were collected from the basolateral side every 10 min, for a total of 70 min, to measure the transport of FITC‐Dextran molecules from the apical to basolateral side. Each withdrawn sample was deposited into a 96 well plate, fluorescence intensity value measured, and the sample then added back to the basolateral side of the culture in order to maintain a constant fluid volume (see below). At the end of performing the transport experiment with 3 kDa FITC‐Dextran, the cell culture inserts were washed with HBSS three times and the transport of 70 kDa FITC ‐Dextran molecules was determined. For each experiment, the concentration of initial stock solutions of 3 kDa and 70 kDa FITC ‐Dextran molecules was determined from calibration graphs. Each sample replicate was individually plotted in order to calculate permeability values; the average permeability of 3‐5 replicates was calculated and presented here.

### Relationship of permeability to FITC‐Dextran concentration

Because there are many published methods used to determine transepithelial permeability (Siflinger‐Birnboim et al. 1987; Artursson 1990; Abbott et al. 1997; Gaillard et al. 2001; Matter and Balda 2003; Balda and Matter 2007; Yuan and Rigor 2010), we briefly derive the relationship between time‐dependent changes in a tracer molecule (e.g., FITC‐Dextran) and the transepithelial permeability used in our measurement. We wish to emphasize that this derivation does not distinguish between transcellular and paracellular transport.

Beginning with the equation for mass flux of a tracer from the apical compartment through a semipermeable surface (area “A”) to the basolateral compartment (Fick's first law) (Fick 1855)

(1) $$\frac{dM_{\mathrm{bl}}}{dt}=P\;A\left[C{\left(t\right)}_{\mathrm{ap}}-C{\left(t\right)}_{\mathrm{bl}}\right]$$

where “*M*_bl_” is the total mass of tracer particles in the basolateral side, “*P*” the permeability (units length/time), *C*(*t*)_ap_ the time‐dependent concentration of tracer particle on the apical side and *C*(*t*)_bl_ the time‐dependent concentration of tracer particle on the basolateral side.

Assuming that the apical and basolateral fluid volumes remain constant, dividing by the basolateral fluid volume *V*_bl_ and using the conservation of mass M_0_ = *C*(*t*)_ap_ *V*_ap_ + *C*(*t*)_bl_ *V*_bl_, we obtain:

(2) $$\frac{dC_{\mathrm{bl}}}{dt}=\frac{P\;A}{V_{\mathrm{bl}}}\left[\frac{M_{0}}{V_{\mathrm{ap}}}-C{\left(t\right)}_{\mathrm{bl}}\frac{V_{\mathrm{ap}}+V_{\mathrm{bl}}}{V_{\mathrm{bl}}}\right]$$

Given the initial condition *C*_bl_(0) = 0, this equation has the solution:

(3) $$C{\left(t\right)}_{\mathrm{bl}}=C_{0}\left[1-\mathrm{Exp}\left[-PA\left(\frac{1}{V_{\mathrm{bl}}}+\frac{1}{V_{\mathrm{ap}}}\right)t\right]\right]$$

where C_0_ is the initial tracer concentration *C*_0_ = *M*_0_/(*V*_ap_+*V*_bl_). For convenience we define the constant *B*  =  *A*(1/*V*_bl_ + 1/*V*_ap_) and rearrange to solve for the permeability:

(4) $$P\;t=-\frac{\text{ln}\left(1-C{\left(t\right)}_{\mathrm{bl}}/C_{0}\right)}{B}$$

Thus, by measuring the basolateral concentration of FITC‐Dextran C(t)_bl_ as a function of elapsed time “*t*”, we obtain the permeability “*P*” as the slope of a best‐fit line. We present our relevant parameter values here in table form: (Table 1).

**Table 1:** Permeability calculation parameters

Parameter	Value
Initial FITC‐Dextran concentration C_0_	Variable
Area of semipermeable filter A	1.13 cm^2^
Volume of fluid in apical chamber V_ap_	100 *μ*L
Volume of fluid in basolateral chamber V_bl_	1 mL
“B” (see equation (4))	12.43 cm^−1^

Using this approach, **t**he permeability of a cell‐free collagen coated cell culture insert was measured to be (1.21 ± 0.46) × 10^−5 ^cm/sec (3 kDa) and (1.01 ± 0.40) × 10^−6 ^cm/sec (70 kDa).

### Measurement of Transepithelial resistance and voltage

Transepithelial voltage and resistance measurements were performed using Endohm chambers (ENDOHM‐24SNAP or ENDOHM‐12) from World Precision Instruments (WPI) connected to an EVOM2 epithelial voltohmmeter from WPI in voltage‐clamp short‐circuit mode. Short circuit equivalent current (*I*_eq_) was calculated from voltage and resistance read outs using Ohm's law, *I*  =  V/R. We report values of *I*_eq_ relative to the control group. More than 95% of *I*_eq_ was inhibited by 10 *μ*mol/L apical amiloride and thus is considered to be proportional to the activity of ENaC in the apical plasma membrane. Before collecting electrophysiological read outs, 100 *μ*L of apical differentiation media was gently added to cell culture inserts in addition to the 100 *μ*L of apical differentiation media already present to ensure electrical contact between the upper electrodes and apical fluid.

### Cobalt chloride treatment

Cobalt chloride (CoCl_2_) hexahydrate (S93179 from Fisher Scientific) was dissolved in 1X Phosphate Buffer Saline (PBS) buffer to make 100 mmol/L CoCl_2_ stock solution, which was further diluted in cell culture media to achieve the desired concentration of 100 *μ*mol/L. Experiments were performed with CoCl_2_ added to apical differentiation media (APDM), basolateral differentiation media (BLDM), or to both APDM and BLDM.

### Cell lysate preparation

Whole cell lysates were prepared using RIPA 1X cell lysis buffer. The 10X RIPA buffer from Cell Signaling Technology (Product no. 9806) was first diluted to prepare 1X buffer. The Halt Protease Inhibitor Cocktail from Thermo Scientific (Product no. 78430) was added at a final concentration of 1X. The RIPA buffer cell lysis protocol from Cell Signaling Technology was followed to prepare whole cell lysates. The EPO levels were compared in the whole cell lysates.

Nuclear extracts were prepared using NE‐PER Nuclear and Cytoplasmic Extraction Reagents from Thermo Scientific (Product no. 78833). The Halt Protease Inhibitor Cocktail was added to during the extraction as instructed by the manufacturer. The protocol from Thermo Scientific was followed to obtain nuclear extracts. The HIF1*α* levels were compared in the nuclear extracts.

The protein concentrations in each sample were determined using Bradford assay using Coomassie Plus Protein Assay Reagent from Thermo Scientific (Product No. 1856210). In each well of the gel same amount (*μ*g) of proteins were loaded. Before loading the samples in the wells of the polyacrylamide gels, sample buffer was added and samples were heated for 6 min at 95°C.

### Western blot

SDS‐PAGE: 12% acrylamide‐bisacrylamide gels and 4% stacking gels were used. The following antibody concentrations were used: mouse monoclonal *β*‐actin (Novus Biologicals# NB600‐501) at a concentration of 1:10,000, mouse monoclonal HIF1*α* (Santa Cruz # sc‐13515) at a concentration of 1:200, mouse monoclonal EPO (Santa Cruz Biotechnology# sc80995) at a concentration of 1:200, mouse monoclonal GAPDH (Santa Cruz Biotechnology# sc‐166545) at a concentration of 1: 1000, rat monoclonal zonula occludin 1 (ZO‐1) (Developmental Studies Hybridoma Bank at University of Iowa# R26.4C) at a concentration of 1:100, mouse monoclonal sodium‐potassium ATPase (NaKATPase) *α*1 subunit (Developmental Studies Hybridoma Bank at University of Iowa# a6F at a concentration of 1:600. Secondary HRP conjugated anti mouse antibody was used at a concentration of 1:2000. To compare *β*‐actin levels in the membrane which were first analyzed for EPO levels, a stripping buffer, Restore Plus Western Blot Stripping Buffer from Thermo scientific (product no. 46430) was used. Antibodies were stripped from the membrane for 10 min, followed by washing with TBST and the membrane was blocked again before addition of primary antibody, that is, monoclonal beta actin antibody. LI‐COR Image Studio™ Software was used to perform densitometric analysis of western blot results.

### MTT cell viability assay

Cell Titer 96 Non‐Radioactive Cell Proliferation Assay (MTT), G4000 (Promega) was used to determine if cell viability was altered by the addition of CoCl_2_. Sample absorbance values were measured at 570 nm using an absorbance plate reader (Perker Elmer Life Sciences/Wallac Victor Multilabel Counter and Wallac 1420 Manager Software). Using trypan blue dye and a hemocytometer, cell enumeration was performed and a calibration graph was generated from cell seeding densities and absorbance values.

### Statistical analysis

Using Microsoft Excel, a type three (two‐sample unequal variance, heteroscedastic) and a two‐tailed Student's *t*‐test was performed between the groups to determine the level of any significance difference. IBM‐SPSS software was used to perform One‐way ANOVA with post hoc Tukey to make comparisons among more than two groups. Standard errors were calculated from standard deviation values and are shown in the graphs.

## Results

### Application of 100 μmol/L CoCl_2_ increases HIF1α levels

**Figure 2:**
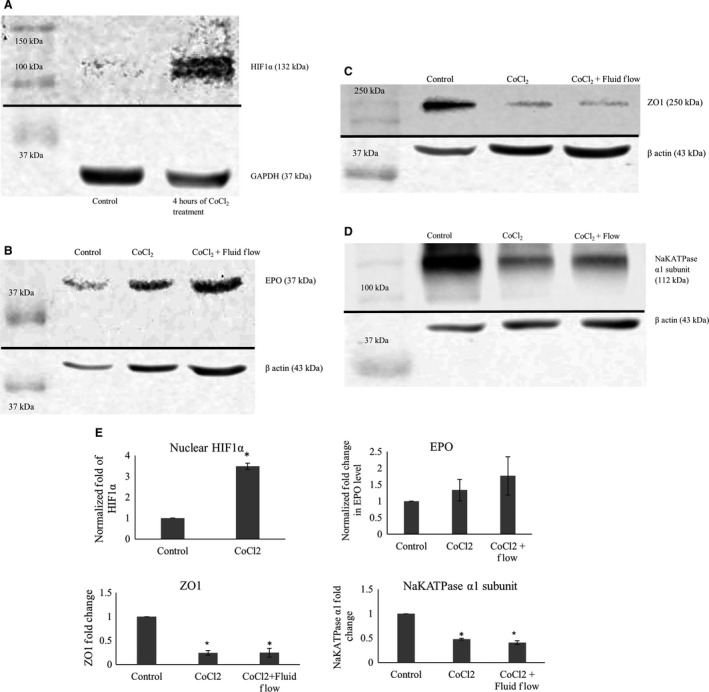
(A) shows western blots of nuclear extracts of mCCD cells treated with 100 *μ*mol/L CoCl_2_. Treatment with CoCl_2_ increased stabilization of HIF1*α* in the nucleus of the cells. GAPDH levels are shown as loading controls. (B) shows western blots of whole cell extracts of mCCD cells treated with 100 *μ*mol/L CoCl_2_ for 4 h. As shown in the images, treatment with CoCl_2_ increased the EPO production by cells. Beta actin levels are shown as loading controls. (C) shows western blots of whole cell extracts of mCCD cells treated with 100 *μ*mol/L CoCl_2_ for 4 h and chronic fluid flow. As shown in the images, treatments decreased ZO‐1 production by cells independently of fluid flow stimulation. Beta actin levels are shown as loading controls. (D) shows western blots of whole cell extracts of mCCD cells treated with 100 *μ*mol/L CoCl_2_ for 4 h and chronic fluid flow. As shown in the images, treatments decreased NaKATPase production by cells independently of fluid flow stimulation. Beta actin levels are shown as loading controls. (E) Densitometric analysis of Western blots presented in (A–D). HIF1*α* level: *P* = 0.004 (*N* = 3), t‐test, showing significant nuclear stabilization of HIF1*α*. EPO level: increases slightly though not statistically significant (*N* = 3, *P* > 0.05, One‐way ANOVA). ZO‐1 level: *P* < 0.0001 (*N* = 4), One‐way ANOVA, showing significant decrease in ZO‐1 levels, independent of fluid flow. NaKATPase *α*1 subunit level: *P* = 0.001 (*N* = 3), One‐way ANOVA, showing significant decrease in NaKATPase *α*1 subunit levels, largely independent of fluid flow.

We performed western blots to demonstrate that addition of 100 *μ*mol/L CoCl_2_ to our culture media significantly increased nuclear HIF‐1*α* and slightly increased HIF‐1*α* effector molecule erythropoietin (EPO) in the whole cell extracts. The findings are in agreement with prior reports (Fisher and Langston 1967; Fandrey et al. 1997; Verghese et al. 2011). Stabilization of HIF1*α* resulted in significant decrease in the levels of ZO‐1 and NakATPase, *α*1 subunit. Western blots and densitometric analysis are shown in Figure 2.

### Effect of Cobalt Chloride on cell viability

The MTT (MTT 3‐(4,5‐dimethylthiazol‐2‐yl)‐2,5‐diphenyltetrazolium bromide) assay is a colorimetric assay for assessing cell metabolic activity and reflects the number of viable cells present (Meerloo et al. 2011). Growing cells, being more active, have higher mitochondrial dehydrogenase activity and show higher MTT absorbance values.

**Figure 3:**
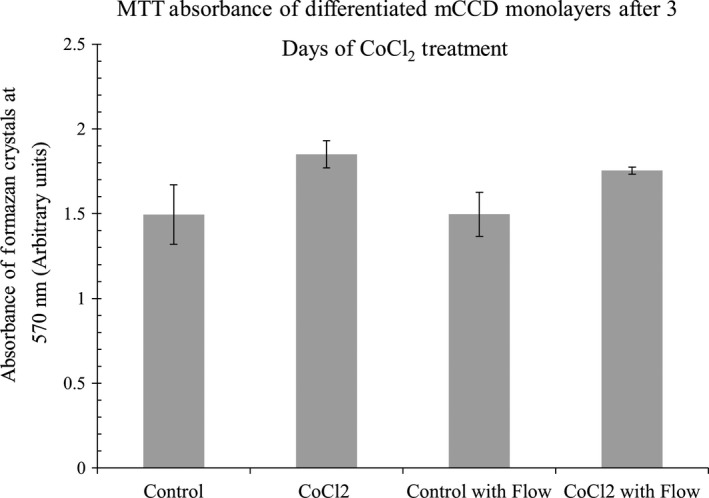
Shows that MTT absorbance values do not significantly change with addition of CoCl_2_, indicating cell viability is unchanged due to 3 days of treatment, either in the presence or absence of fluid flow. *N* = 4–5. One Way ANOVA, non‐significant, *P* > 0.05.

As shown in Figure 3, MTT absorbance values showed that there were no significant differences between control and CoCl_2_ treatment groups either in the presence or absence of fluid flow, indicating that addition of 100 *μ*mol/L CoCl_2_ to our culture media did not affect cellular viability.

### Transepithelial Permeability

**Figure 4:**
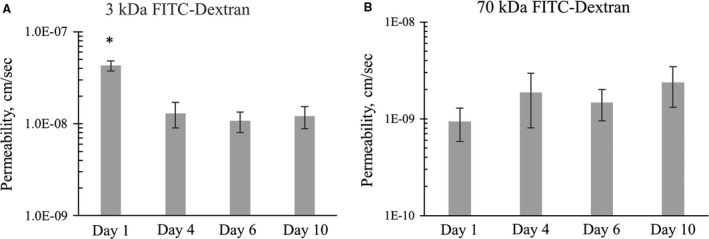
(A) Shows that 3 kDa FITC dextran transepithelial permeability at the onset of differentiation (day 1) is significantly higher as compared to fully differentiated mCCD monolayers (day 10). *N* = 3–6. One‐way ANOVA *P* < 0.002. (B) shows that the permeability of 70 kDa FITC‐dextran does not change significantly during differentiation. *N* = 3–6, One‐way ANOVA *P* > 0.05.

As mCCD epithelial cells differentiate, they form tight junctions and tissue‐level gain‐of‐function properties such as directed transport of salt and water begin to emerge. Figure 4A presents transepithelial permeability data of mCCD monolayers during this differentiation process. As expected, permeability at day 1 was significantly higher as compared to fully differentiated mCCD monolayers after 10 days of differentiation. This measurement provides some evidence that the principal diffusion path for 3 kDa FITC‐Dextran in mCCD cells may be via paracellular route, as tight junctions form early in the differentiation process. In contrast, the permeability of 70 kDa FITC‐Dextran showed no changes during differentiation (Fig. 4B). During later phase of differentiation 3 kDa and 70 kDa permeability values were stable.

**Figure 5:**
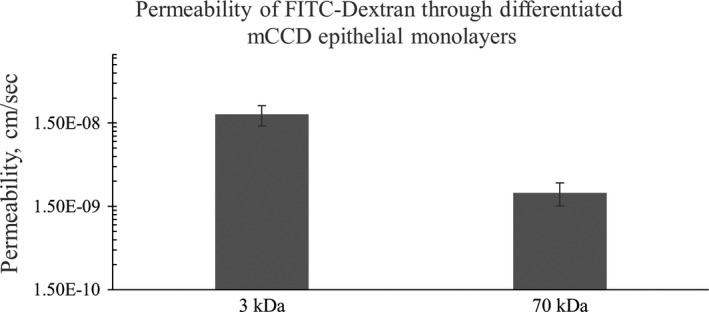
Shows the transepithelial permeability of fully differentiated mCCD monolayers. *N* = 8. T‐test: *P* value < 0.02.

Once fully differentiated, mCCD monolayers showed 3 kDa FITC‐Dextran permeability of (1.9 ± 1.5) × 10^−8 ^cm/s and 70 kDa FITC‐Dextran permeability of (2.2 ± 1.9) × 10^−9 ^cm/sec (Fig. 5). Both of these are significantly reduced as compared to the cell‐free insert, demonstrating the efficacy of tight junctions in regulating transcellular transport.

### CoCl_2_ increases monolayer permeability:

**Figure 6:**
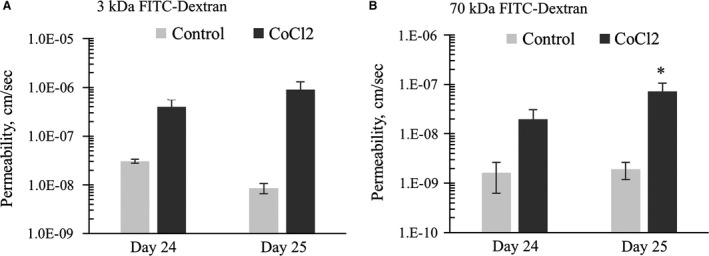
(A) CoCl_2_ added on day 22. Data shows that CoCl_2_ treatment for 48 h (Day 24) and 72 h (Day 25) increased the permeability of differentiated mCCD monolayers measured with 3 kDa FITC‐dextran. *N* = 4–5, One‐way ANOVA. CoCl_2_ treatment significantly (*P* value = 0.02) increases monolayer permeability on day 25. No other statistical significant difference was noticed among any other groups. (B) CoCl_2_ added on day 22. Data shows that CoCl_2_ treatment for 3 days increased the permeability of differentiated mCCD monolayers measured with 70 kDa FITC‐dextran. *N* = 4–5, One‐way ANOVA. CoCl_2_ treatment significantly (*P* value = 0.02) increases monolayer permeability on day 25. No other statistical significant difference was noticed among any other groups.

We investigated the effect of CoCl_2_ in altering monolayer permeability. Addition of 100 *μ*mol/L CoCl_2_ to fully differentiated monolayers for 48 h increased 3 kDa FITC‐Dextran permeability to (4.02 ± 3.15) × 10^−7 ^cm/s and addition of 100 *μ*mol/L CoCl_2_ for 72 h increased the permeability further to (9.05 ± 8.13) × 10^−7 ^cm/s (Fig. 6A). Similarly, the transepithelial permeability of 70 kDa FITC‐Dextran was (1.99 ± 2.13) × 10^−8 ^cm/s at 48 h and (7.24 ± 6.47) ×10^−8 ^cm/s at 72 h (Fig. 6B). That is, CoCl_2_ increased 3 kDa dextran‐fluorescein permeability more than 100‐fold and 70 kDa dextran‐fluorescein permeability nearly 40‐fold. In conclusion, CoCl_2_ was found to significantly increase the transepithelial permeability of FITC‐Dextran molecules through fully differentiated mCCD monolayers.

## Transepithelial electrophysiology Results:

### Transepithelial resistance

**Figure 7:**
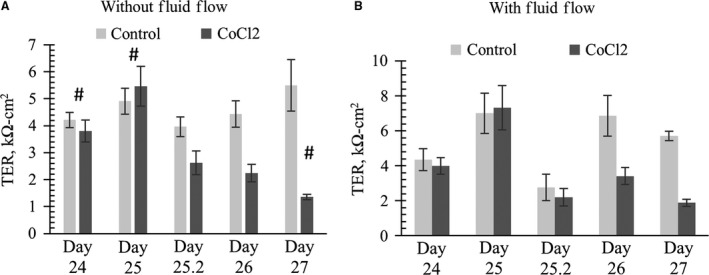
CoCl_2_ added to mCCD cultures on day 24 in the absence (A) or presence (B) of chronic steady flow. *N* = 4–16. Data shows that application of 100 *μ*mol/L CoCl_2_ significantly decreased TER values of differentiated mCCD monolayers after 48 h (2 days) without fluid flow. One‐way ANOVA shows no significant (*P* > 0.05) TER changes among control groups in (A) or (B). ‘*’ denotes significant change (*P* < 0.05) between a control and CoCl_2_ treatment at a specific time‐point. ‘#’ denotes significant change (*P* < 0.05) between CoCl_2_‐treated sample at one time point with CoCl_2_‐treated or control sample at other time points. No statistical difference was found between CoCl_2_ treatment on day 26 and day 27 in (A). In figure (B) with fluid flow, at a specific time point, statistical significance (*P* > 0.05) was not detected between control and CoCl_2._ However, we noticed CoCl_2_ treatment group value was significantly decreased as compared to control value at a different time point (*P* < 0.05, denoted by #). TER on day 27 of CoCl_2_‐treated group was significantly decreased as compared to Day 26 control. Similarly, TER on day 26 of CoCl_2_‐treated group was significantly decreased as compared to Day 25 control.

Due to tight junctions, an untreated confluent and fully differentiated mCCD monolayer has a transepithelial resistance (TER) value around 3 kΩ‐cm^2^. As shown in Figure 7A and B, extended application of CoCl_2_ results in a significant decrease in the TER value at day 2 and 3, consistent with the measured increase in transepithelial permeability. After 72 h with CoCl_2_ treatment, the TER of mCCD monolayers were found to decrease fourfold in the absence of fluid flow and threefold in the presence of fluid flow. Statistical analysis shows that the effect of HIF stabilization in decreasing monolayer resistance was enhanced when fluid flow was absent.

### Transepithelial Equivalent short‐circuit current *I*_eq_

**Figure 8:**
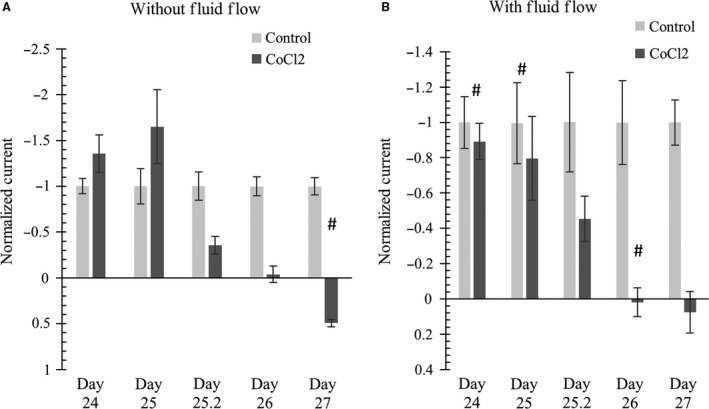
(left) without applied flow; (right) with applied flow. CoCl_2_ added on day 24. Data shows that application of 100 *μ*mol/L CoCl_2_ significantly decreased *I*_eq_ values of differentiated mCCD monolayers after 48 h (2 days). Interestingly, after 3 days of treatment, *I*_eq_ value switched to the positive direction. Current values are plotted as relative to average *I*_eq_ values as discussed in the main text. *N* = 4–16. One‐way ANOVA shows no significant (*P* > 0.05) *I*_eq_ changes among control groups in (A) or (B). * denotes significant change (*P* < 0.05) between a control and CoCl_2_ treatment at a specific time‐point. # denotes CoCl_2_ treatment causes significant change (*P* < 0.05) between CoCl_2_‐treated sample at a specific time point with CoCl_2_‐treated or control sample at a different time point. In Figure (A) and (B), CoCl_2_ treatment shows gradual decrease in negative *I*_eq_; some of this gradual decrease was statistically significant (e.g., Figure (A) Day 25 CoCl_2_ vs. Day 26 CoCl_2_).

Figure 8 shows that the effect of CoCl_2_ on *I*_eq_ measurements was more prominent in the absence of fluid flow as compared to monolayers cultured in the presence of fluid flow. In response to CoCl_2_ treatment, significant decreases of *I*_eq_ values were found after 30 h in the absence of flow (Fig. 8A). In response to CoCl_2_ treatment for 3 days in monolayer cultures not exposed to fluid flow, *I*_eq_ values showed a significant change and, very significantly, a positive mean *I*_eq_value of 0.9^^ *μ*A/cm^2^ was developed. Movement of positive sodium ions from the apical to basolateral side results in a negative *I*_eq_ value. Thus, a positive *I*_eq_ value implies that net ion transport may be moving in the opposite direction. It is tempting to interpret these results as confirmation of our hypothesis that increased levels of HIF can transform a normally absorptive epithelial monolayer into a secretory monolayer.

Comparison of normalized *I*_eq_ values of mCCD monolayers cultured with chronic fluid flow showed that *I*_eq_ values significantly decreased at day 2 in response to CoCl_2_ treatment as shown in Figure 8B. Control samples had a mean *I*_eq_ value of −2.6 *μ*A/cm^2^_,_ whereas CoCl_2_‐treated samples showed mean *I*_eq_ values of 0.1 *μ*A/cm^2^ (48 h) and 0.2 *μ*A/cm^2^ (72 h).

Comparing Figures 8A and B, it should be noted that CoCl_2_ altered *I*_eq_ values in the monolayers without fluid flow were significantly altered after 30 h, whereas in the monolayers cultured with fluid flow, significant changes occurred after 48 h. Therefore, it can possibly be inferred that the lack of fluid flow can intensify the effect of HIF stabilization.

### CoCl_2_ Acts on Basolateral side

**Figure 9:**
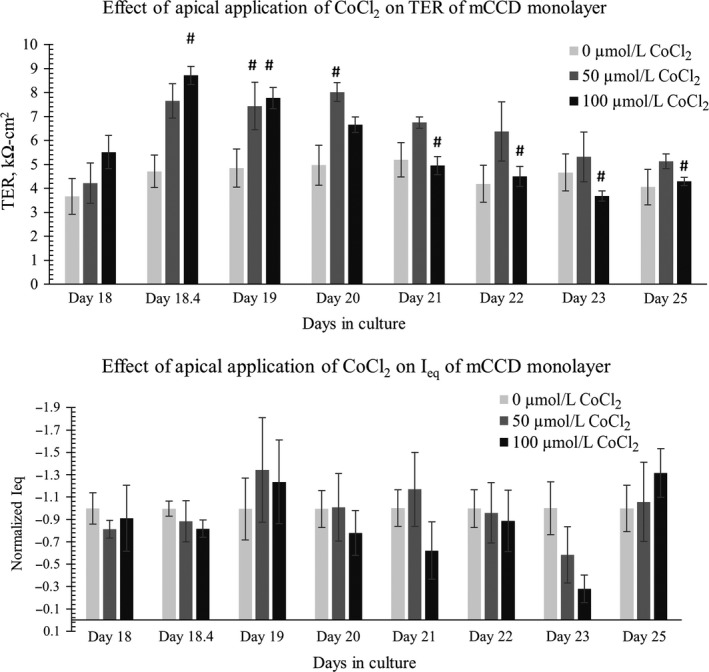
CoCl_2_ added to apical media on day 18. Data shows that application of CoCl_2_ in apical media does not cause a significant chronic change in (top) TER values or (bottom) *I*_eq_ values of differentiated mCCD monolayers. *N* = 5–8. In the TER and *I*_eq_ data, One‐way ANOVA shows no significant (*P* > 0.05) TER and I_eq_ changes among control groups. In the TER plot, * denotes significant change (*P* < 0.05) among 0 *μ*mol/L CoCl_2_, 50, and *μ*mol/L 100 CoCl_2_ treatment at a specific time‐point. # denotes CoCl_2_ treatment causes significant change (*P* < 0.05) between CoCl_2_‐treated sample at a specific time point with CoCl_2_‐treated or control sample at a different time point. Not all significance (#) are shown in the graph; all # are marked onto any noticeable change and applicable to group treated with CoCl_2_. There is no significant effect (*P* > 0.05) of apical application of 0, 50, or 100 *μ*mol/L CoCl_2_ on *I*_eq_.

**Figure 10:**
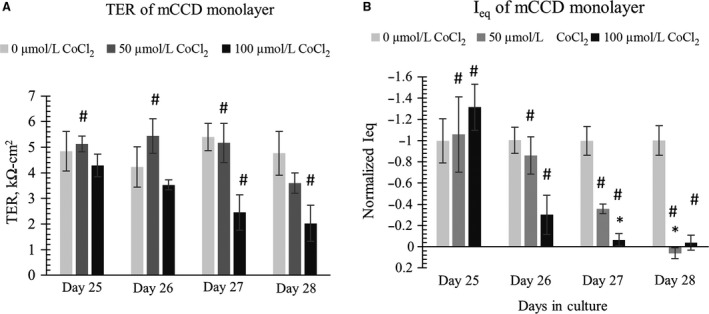
CoCl_2_ was withdrawn from apical media and added to basolateral media on day 25. Data shows that application of 100 *μ*mol/L CoCl_2_ to basolateral media significantly decreased (left) TER values and (right) *I*_eq_ values of differentiated mCCD monolayers in a dose‐dependent manner after 2 days of treatment (*N* = 5–6). In the TER and *I*_eq_ data, One‐way ANOVA shows no significant (*P* > 0.05) TER and *I*_eq_ changes among control groups. In the TER plot, * denotes significant change (*P* < 0.05) among 0 *μ*mol/L CoCl_2_, 50, and *μ*mol/L 100 CoCl_2_ treatment at a specific time‐point. # denotes CoCl_2_ treatment causes significant change (*P* < 0.05) between CoCl_2_‐treated sample at a specific time point with CoCl_2_‐treated or control sample at a different time point. In figure (A) we do not see statistically significant decrease in TER, * (*P* < 0.05) on day 27 or day 28 by 50 or 100 *μ*mol/L CoCl_2_ as compared to control (0 *μ*mol/L CoCl_2)_ on that specific day. However, we notice some significant decrease in TER as compared to control at a different time point. For example on day 28, 100 *μ*mol/L CoCl2 causes significant decrease in the TER value when compared to 0 *μ*mol/L CoCl2 TER value on day 27, denoted by #. In figure (B) we notice significant loss of *I*_eq_ with 100 *μ*mol/L CoCl_2_ treatment on day 27 and significant loss of *I*_eq_ with both 50 and 100 *μ*mol/L CoCl_2_ treatment on day 28. Not all significance (#) are shown in the graph; all # are marked onto any noticeable change and applicable to group treated with CoCl_2_.

In our cell lines, the transporter responsible for couptake has not been clearly identified. In mammalian cells, the divalent metal cation transporters Slc11a1 and Sla11a2 may be responsible for couptake (Gunshin et al. 1997; Forbes and Gros 2003). We found that mCCD monolayers cultured in flow exposed to apical CoCl_2_ treatment showed an initial, transient, increase in TER but no chronic change to the TER. The *I*_eq_ values mostly remained unaffected except for a transient change on day 23 in culture (i.e., 5 days of CoCl_2_ treatment), shown in Figure 9. However, when CoCl_2_ was added only to the basolateral media, we noted significant dose‐dependent decreases in both TER and *I*_eq_ values, shown in Figure 10. Basolateral treatment with 100 *μ*mol/L CoCl_2_ showed significant decreases in TER values after 2 days of treatment as shown in Figure 10A. Thus, it is interesting to find that CoCl_2_ affects the electrophysiological properties of our monolayers only when it is added in the basolateral media, potentially identifying the site of couptake.

## Discussion

### 100 μmol/L CoCl_2_ stabilizes HIF‐1α without affecting cell viability

In our mCCD monolayers 100 *μ*mol/L CoCl_2_ was found to significantly stabilize HIF‐1*α* in the nucleus and also resulted in a slight increased production of EPO, not statistically significant. 100 *μ*mol/L CoCl_2_ was previously reported to stabilize HIF1*α* in the nucleus of Madin‐Darby Canine Kidney (MDCK) epithelial cells (Verghese et al. 2011). Other studies showed cobalt can increase EPO production (Fisher and Langston 1967; Fandrey et al. 1997). CoCl_2_ was previously shown to prevent cell growth of mouse embryonic fibroblasts at a concentration of 100 *μ*mol/L. However, our MTT assay showed 100 *μ*mol/L CoCl_2_ did not have any significant effect on the cell viability of the fibroblasts after 72 h (Vengellur and LaPres 2004). Similarly, we did not notice any change in the cell viability levels of mCCD kidney epithelial cells by 100 *μ*mol/L CoCl_2_.

As mentioned previously, in kidney cystic epithelia of rats and humans HIF‐1*α* protein level and mRNA levels of HIF*α* target genes, such as EPO, glucose transporter‐1 (Glut‐1), and vascular endothelial growth factor (VEGF) were found to be elevated (Bernhardt et al. 2007). Using CoCl_2_ we stabilized HIF‐1*α* in our kidney epithelial monolayers to better understand potential roles of ischemia‐hypoxia or otherwise HIF stabilized environment as experienced by normal kidney epithelial cells in an ischemic environment created by the presence of cysts in PKD.

### Effect of Fluid Flow with HIF stabilization

Our electrophysiological results suggest that the presence of fluid flow may blunt HIF‐stabilized altered transepithelial transport. For example, both *I*_eq_ and TER values decreased more rapidly in the absence of fluid flow as compared to monolayers cultured in the presence of chronic fluid flow.

While it is tempting to suggest that monolayers cultured in the absence of fluid flow may be hypoxic to some extent and may already have increased HIF to some extent, our use of thin apical fluid thickness ensures adequate O_2_ transport to the monolayer. Two possible alternative explanations for more prominent effects of CoCl_2_ in cultures lacking fluid flow are (1) the level of stabilized HIF*α* by CoCl_2_ may be higher in cultures lacking fluid flow, or (2) CoCl_2_ can stabilize HIF*α* in the cultures without fluid flow at an earlier time point as compared to cultures with fluid flow. We tried to directly measure alterations of HIF levels via Western blot, but our bands were too faint to provide meaningful results. We compared EPO levels but could not detect any statistically significant difference between CoCl_2_ treated with or without fluid flow. Moreover, comparing ZO‐1 and NaKATPase *α*1 subunit levels, we could not detect any statistically significant difference due to HIF‐1*α* stabilization with or without fluid flow. Taken together, this implies that the examined epithelial response to fluid flow is independent of HIF‐1*α*.

### HIF Stabilization by CoCl_2_ alters transcellular and paracellular transport

In the mammalian kidney, paracellular permeability is known to decrease from proximal tubule to collecting duct due to the unique expression of claudins and higher levels of occludin and zonula occludin‐1 (ZO‐1) (Gonzalez‐Mariscal et al. 2000; Denker and Sabath 2011). Paracellular transport through epithelial tight junctions is driven by the ion gradient created due to transcellular active transport. In our mCCD monolayers HIF stabilization for 72 h with CoCl_2_ resulted in a 100 fold increase in 3 kDa FITC‐dextran permeability and 40 fold increase in 70 kDa FITC ‐dextran permeability. Thus, paracellular transport through tight mCCD monolayers was found to increase due to HIF stabilization.

Epithelial tight junctions maintain the high electrochemical gradient created by transcellular transport (Anderson and Van Itallie 2009). High TER values generally correspond to low paracellular flux and low TER values generally refer to monolayers with high paracellular flux. mCCD monolayers show high TER values, around 3 kΩ‐cm^2^. Increased FITC‐dextran permeability is consistent with decreased TER values due to HIF stabilization. The cortical collecting duct is responsible for 0.5% salt reabsorption and helps in fine‐tuning water and salt balance in the body. mCCD *I*_eq_ is primarily generated by sodium transport through ENaC channels and 95% of *I*_eq_ can be blocked by 10 *μ*mol/L amiloride, a sodium channel blocker (Resnick and Hopfer 2007; Resnick 2011). We noticed that HIF stabilization by CoCl_2_ caused decreased *I*_eq_ values, indicating mainly the loss of active sodium transport.

Similar observations to our results were made studying the effect of hypoxia on aleveolar epithelial cells (Mairbäurl et al. 2002). Hypoxia was found to decrease active sodium transport through rat alveolar epithelial monolayers and also decreased TER of monolayers, increasing permeability of 4 kDa dextran‐FITC through the epithelial monolayers (Mairbäurl et al. 2002; Bouvry et al. 2006). Hypoxia was found to decrease ZO‐1 protein levels in the same alveolar monolayer (Bouvry et al. 2006). Hypoxia can affect the actin cytoskeleton organization and can result in occludin mislocalization from tight junctions to the cell interior (Bouvry et al. 2006). Hypoxia was shown affect the expression or activity of apical ENaC and basolateral NaKATPase causing decrease in active sodium transport, resulting in reduced ability of alveolar epithelium to clear alveolar edema fluid (Vivona et al. 2001; Planès et al. 2002; Dada et al. 2003). Hypoxia was shown to alter tight junctions and permeability of other cell types, such as brain microvessel endothelial cells and intestinal epithelial cells (Xu et al. 1999; Yamagata et al. 2004; Engelhardt et al. 2014), supporting our findings that HIF stabilization can decrease monolayer resistance and can increase FITC‐dextran permeability. Similarly, our western blots showed that stabilization of HIF1*α* by CoCl_2_, decreased levels of ZO‐1 and NaKATPase *α*1 subunit independent of fluid flow.

Our measurements demonstrating altered transepithelial permeability is relevant is supported by other results demonstrating that epithelial tight junction morphology is regulated by HIF, with claudins (Saeedi et al. 2015) and occludins (Caraballo et al. 2011) being specific targets. Similarly, our electrophysiological measurements are in concordance with other results (Caraballo et al. 2011).

Stabilization of HIF1*α* by CoCl_2_ results in decreased TER values of mCCD monolayers corresponding to increased permeability of 3 kDa and 70 kDa dextran‐fluorescein. 3 kDa dextrans can be transported through epithelial monolayers either by fluid‐phase transcytosis or by paracellular transport, whereas large 70 kDa dextran molecules are known to be transported only through the paracellular pathway (Matter and Balda 2003). Thus, changes in 70 kDa transport through mCCD monolayers represent changes in the paracellular pathway.

Using 3 kDa and 70 kDa FITC‐Dextran molecules, we were able to identify that the size selectivity was maintained by mCCD epithelial monolayer tight junctions. Increased permeability of anionic FITC‐dextran from apical to basolateral side suggests increased paracellular transport which can also be indicative of increased water permeability.

## Conclusion

In summary, in mCCD monolayers, we noted that HIF stabilization by CoCl_2_ caused loss of *I*_eq_ and may show a positive *I*_eq_ value in the absence of fluid flow, indicating that the net flux of sodium ions may be transported in the opposite direction and thus those epithelial monolayers may be secretory rather than absorptive. In mCCD monolayers HIF stabilization showed a significant decrease in the TER values and increase in 70 kDa permeability due to paracellular pathway changes. Thus, HIF stabilization seems to affect both the transcellular and paracellular pathways of kidney epithelial monolayers. Increased FITC‐dextran permeability through cellular monolayers of mCCD indicated altered selective epithelial barrier function. Decreased levels of tight junction protein, such as ZO‐1 causes decreased resistance and increased paracellular permeability of epithelial monolayer. Decreased levels of basolateral enzyme NaKATPase can be at least partly responsible for the loss of transcellular transport of sodium ions. Moreover, we identified that CoCl_2_ uptake may occur via basolateral side of monolayers.

In conclusion, we have provided preliminary evidence in support of our hypothesis that HIF stabilization can contribute to kidney cyst expansion by increasing tissue permeability and reversing net sodium transport through normal kidney epithelia. We suspect this path may also involve cAMP, as increased levels of cAMP are associated with microcysts. It has been shown that HIF‐1*α* contains a cAMP‐response element binding (CREB) binding site (Kvietikova et al. 1995). It has also been shown that HIF‐1*α* accumulates via the cAMP‐mediated induction of the mTOR pathway in beta cells (Van de Velde et al. 2011), but we did not directly test the role of cAMP in our cells.

## Conflict of Interest

The authors have no conflicts of interest to declare.
